# High prevalence of factor XIII deficiency and its association with bleeding in extracorporeal membrane oxygenation patients: an observational study from a prospectively compiled biobank

**DOI:** 10.1016/j.rpth.2025.103254

**Published:** 2025-11-19

**Authors:** Andrea Kornfehl, Roman Brock, Peter Quehenberger, Harald Herkner, Michael Schwameis, Peter Schellongowski, Bernhard Nagler, Markus Eder, Thomas Staudinger, Nina Buchtele

**Affiliations:** 1 Department of Medicine I, Intensive Care Unit 13i2; Medical University of Vienna, Vienna, Austria; 2 Department of Emergency Medicine, Medical University of Vienna, Vienna, Austria; 3 Department of Laboratory Medicine, Medical University of Vienna, Vienna, Austria

**Keywords:** blood coagulation, blood coagulation tests, extracorporeal life support, extracorporeal membrane oxygenation, hemostasis, thromboembolism

## Abstract

**Background:**

Coagulopathy is a major cause of death during extracorporeal membrane oxygenation (ECMO). Factor (F)XIII may play an important role in bleeding risk and represents a potential target for future therapies.

**Objectives:**

In order to explore the potential of using FXIII as a therapeutic target in ECMO, we assessed its activity, identified the risk factors associated with deficiency and evaluated its link to bleeding complications.

**Methods:**

This observational study investigated FXIII activity in adult ECMO patients using prospectively collected blood samples. The primary outcome was the prevalence of FXIII deficiency (<70%). Secondary outcomes included the incidence of bleeding complications according to the Bleeding Academic Research Consortium classification. Regression models were used to analyze risk factors for FXIII deficiency and its association with bleeding.

**Results:**

Between March 2020 and September 2023, FXIII activity was assessed in 101 ECMO patients undergoing a total of 108 ECMO runs. FXIII deficiency was identified in 73.3% (*n* = 74) of patients and occurred more frequently in those with viral infections, prolonged ECMO duration, and venovenous ECMO support. Bleeding complications were more frequent in patients with FXIII deficiency (41.9% vs 11.1%; *P* = .003), and the median FXIII activity was significantly associated with bleeding complications (odds ratio, 0.97 per 1% increase in activity; 95% CI, 0.94-0.99; *P* = .003). Similarly, minimum FXIII activity was independently associated with bleeding risk (odds ratio, 0.967; 95% CI, 0.93-0.99; *P* = .04).

**Conclusion:**

In this cohort of adult ECMO patients, we observed a high prevalence of FXIII deficiency. FXIII deficiency was common in adult ECMO patients and associated with viral infections, venovenous ECMO, longer ECMO duration, and bleeding; its therapeutic potential warrants further study.

## Essentials

• Bleeding is a major challenge in ECMO, with FXIII impacting hemostasis.
• We conducted a prospective study of FXIII deficiency prevalence, bleeding, and risk factors in ECMO patients.
• Bleeding complications were markedly more frequent in ECMO patients with FXIII deficiency.
• FXIII activity was found to be an independent predictor of bleeding risk.

## 1 Introduction

Extracorporeal membrane oxygenation (ECMO) has emerged as a rescue therapy for both circulatory and respiratory failure [[1], [2], [3]], driven in part by lower complication rates with newer devices and increasing expertise among providers.

Bleeding and thrombotic complications remain major causes of mortality in ECMO patients, with coagulation factor (F)XIII potentially playing a key role in clot stability [[4], [5], [6]].

In surgical populations, reduced FXIII activity has been linked to increased perioperative blood loss, transfusion needs, and surgical reexploration [7]. A recent retrospective cohort study [8] of ECMO patients with high bleeding risk reported frequent FXIII deficiency. However, data from prospectively collected, consecutive patients are currently lacking, and therefore, a definitive association with bleeding complications remains to be established [[9], [10], [8]]. To explore FXIII as a potential therapeutic target in ECMO, we assessed FXIII activity, identified risk factors for its deficiency, and evaluated its association with bleeding complications. We hypothesized that FXIII deficiency is common in ECMO patients and that lower FXIII activity levels are associated with an increased risk of bleeding complications.

## 2 Methods

### 2.1 Study design and patients

This study was an observational study using prospectively collected samples from venovenous (VV) and venoarterial ECMO patients (1898 in 2017 and 2329 in 2019). The study coordination and sample acquisition were conducted at a medical intensive care unit (ICU) at a European tertiary care center. Only adult patients (≥18 years) were included. Blood samples were obtained twice within the first week of ECMO and once weekly thereafter. Citrated blood was centrifuged at 2000 × *g* for 10 minutes, and the plasma was stored at −80 °C until analysis. FXIII activity (%) was determined by a standardized coagulometric method with photoptic detection (normal range, 70%-140%) [[11], [12], [13], [14]]. The activity of FXIII in citrated plasma was measured using the automated World Health Organization-standardized Berichrom FXIII chromogenic assay (Siemens Healthineers) on a Sysmex CS-2500 analyzer. Other coagulation factors’ (FII, FV, FVII, FVIII, FIX, FX, FXI, and FXII) activity (%) and lupus anticoagulant were determined during clinical routine in cases of discrepancy between antiXa and activated partial thromboplastin time. Thromboplastin time (%), activated partial thromboplastin time (seconds), thrombin time (seconds), fibrinogen (Clauss method, mg/dL), and antithrombin (%) were measured at least twice daily.

All patients received anticoagulation with an unfractionated heparin bolus upon cannulation, followed by continuous administration of unfractionated heparin (maintenance dose of 5-10 IU/kg/h). Patients with heparin-induced thrombocytopenia type II received argatroban. Anticoagulation was monitored by antiXa (antiXa target 0.2-0.3 IU/mL). Bleeding events were assessed daily and reported following the Bleeding Academic Research Consortium bleeding classification [15]. Thromboembolism was defined as membrane occlusion related to thrombosis or pump thrombosis necessitating system exchange, as well as thrombus formation in the cardiovascular system.

The study was reported to the Ethics Committee of the Medical University of Vienna (institutional review board reference numbers 1898/2017 and 2329/2019). Each surviving patient signed an informed consent form following treatment.

### 2.2 Outcomes

The primary outcome was the prevalence of FXIII deficiency, which was defined as a FXIII activity level below 70% (the median FXIII activity level was calculated from all measurements obtained during ECMO support). The secondary outcomes included the incidence of bleeding complications in the presence or absence of FXIII deficiency, as well as the identification of potential risk factors for FXIII deficiency.

### 2.3 Statistical analysis

Descriptive statistics were calculated to describe the prevalence of FXIII deficiency and the trend of FXIII activity during and after ECMO support. Comparative analyses were conducted between patients with and without FXIII deficiency with regard to the following outcome parameters: ECMO duration, ICU and hospital survival, thromboembolic/bleeding complications, and underlying conditions such as pneumonia and extracorporeal cardiopulmonary resuscitation. The Mann–Whitney U-test was used for nonnormally distributed continuous or ordinal variables, and the chi-squared test for categorical variables. Group comparisons were performed to identify risk factors for FXIII deficiency. Variables included C-reactive protein, the liver sequential organ failure assessment score domain, D-dimer, and disseminated intravascular coagulation score. For these analyses, laboratory values from the first day after ECMO initiation were used. Logistic regression analysis was performed to evaluate risk factors for bleeding complications. The assumption of a linear trend across FXIII tertiles was evaluated using a likelihood-ratio test. Variables included in the model, chosen based on clinical plausibility, were FXIII activity, age, platelet count, fibrinogen levels (mg/dL), and ECMO runtime. Bleeding-free survival was estimated using the Kaplan–Meier method and the log-rank test. Time to bleeding was defined as the number of days from ECMO initiation to the first documented bleeding event. For all analyses, only the first bleeding event per patient was considered. The FXIII activity measurements included in the regression analysis were restricted to values obtained prior to or at the time of the first bleeding event in order to reflect the temporal association and minimize bias. The level of significance was set at *P* = .05. We used Stata version 16 (StataCorp) and SPSS Statistics for Windows version 27.0 (IBM Corporation) for data management and analyses.

## 3 Results and Discussion

**Figure 1:**
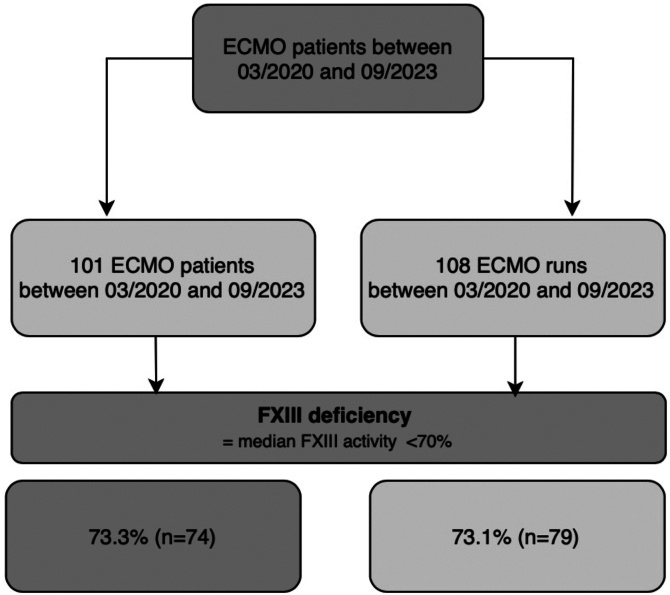
Patient flowchart. ECMO, extracorporeal membrane oxygenation; FXIII, factor FXIII.

Between March 2020 and September 2023, 101 patients were included. Seven of them underwent a second ECMO run. Patients were predominantly male (*n* = 70; 69.3%), with a median age of 56 years (IQR, 13). The median ECMO runtime was 12 days (IQR, 20). Sixty-eight patients (67.3%) were successfully discharged from the ICU, and 66 patients (65.3%) were discharged from the hospital. No patient received a cryoprecipitate or FXIII substitution. Baseline characteristics are shown in Table 1 and Figure 1.

**Table 1:** Baseline characteristics and outcome.

Baseline characteristics	Overall (*N* = 101)	FXIII deficiency (*n* = 74)	No FXIII deficiency (*n* = 27)	*P* values
Age (y), median (IQR)	56 (13.8)	58 (13)	54 (16)	.103
Male, sex	70 (69.3)	49 (66.2)	21 (77.8)	.33
Type of extracorporeal life support				.05
VV ECMO	62 (61.4)	50 (67.6)	12 (44.4)	
VA ECMO	35 (34.7)	20 (27)	15 (55.6)	
Other	4 (4.0)	4 (5.4)	0 (0.0)	
SAPS II at ICU admission, median (IQR)	41 (15.8)	42 (15.5)	40 (17)	.135
**Outcomes**				
ECMO runtime (d)	12.5 (15.8)	14 (24)	5 (11)	**.006**
ICU survival	68 (67.3)	49 (66.2)	19 (70.4)	.81
Hospital survival	66 (65.3)	47 (63.5)	19 (70.4)	.639
Thromboembolism^a^	51 (50.5)	42 (56.8)	9 (33.3)	**.045**
Bleeding^a^	34 (33.7)	31 (41.9)	3 (11.1)	**.003**
**Underlying disease**				**.016**
Viral pneumonia	58 (57.4)	49 (66.2)	9 (33.3)	
COVID-19	48 (47.5)	40 (54.1)	8 (29.6)	
Bacterial pneumonia	5 (5.0)	4 (5.4)	1 (3.7)	
eCPR	26 (25.7)	15 (20.3)	11 (40.7)	
Other	12 (11.9)	6 (8.1)	6 (22.2)	

Data are *n* (%) unless otherwise specified. *P* values represent between-group comparisons of patients with and without FXIII deficiency.

ECMO, extracorporeal membrane oxygenation; eCPR, extracorporeal cardiopulmonary resuscitation; FXIII, factor XIII; ICU, intensive care unit; SAPS II, Simplified Acute Physiology Score II; VA, venoarterial; VV, venovenous.

a Numbers display patients with at least 1 thromboembolic/bleeding event.

**Figure 2:**
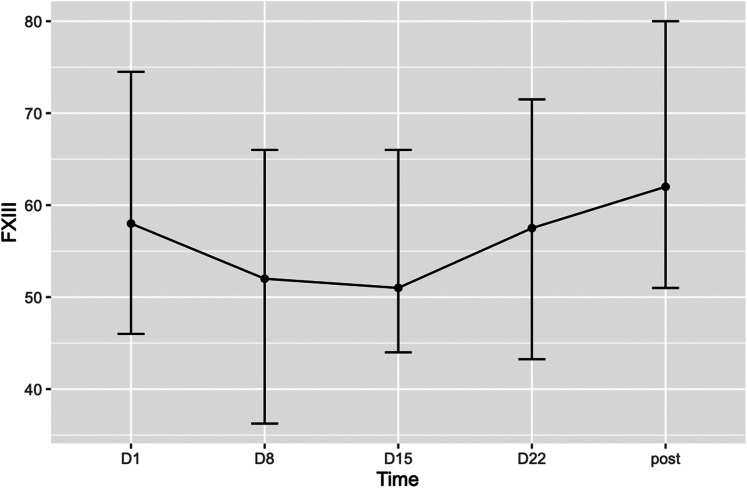
Factor (F)XIII activity over the course of extracorporeal membrane oxygenation support. The levels of FXIII activity are shown at predefined time points (day [D]1, D8, D15, D22, and posttreatment). Error bars represent the corresponding SDs. FXIII activity initially declined during the early treatment phase, before recovering toward posttreatment levels.

Across all ECMO runs, the median FXIII activity (the median FXIII activity level was calculated from all measurements obtained during ECMO support) was 58% (IQR, 29.0) on days 1 to 3, 52% (IQR, 31.8) on days 8 to 15, 51% (IQR, 25.0) on days 15 to 21, and 58% (IQR, 30.0) on days 22 to 29. Consequently, the median FXIII activity of 58% on ECMO day 1 increased to 62% (IQR, 29.0) after decannulation of ECMO. When considering only the first ECMO run per patient, the results were similar (58%; IQR, 24.5). No significant decrease in FXIII activity was observed over the course of ECMO support (−0.087% per ECMO day; *P* = .61; 95% CI, −0.42 to 0.25). FXIII activity increased after ECMO removal (*P* value = .41 and .207). Further details are provided in Table 2 and Figure 2.

**Table 2:** Median factor XIII activity over the course of treatment.

	D1	D8	D15	D22	Post
Overall (*N* = 108)					
FXIII activity (%), median (IQR)	58 (29.0)	52 (31.0)	51 (25.0)	58 (30.0)	62 (29.0)
No. of samples, (%)	103 (95.4)	60 (55.6)	37 (34.3)	26 (24.1)	77 (71.3)
ECMO blood sampling day, mean (SD)	1.4 (1.2)	8.8 (1.5)	15.9 (1.2)	23 (1.7)	20 (16.8)
First EMCO run (*n* = 101)					
FXIII activity (%), median (IQR)	58 (27.8)	52 (31.8)	51 (28.0)	57 (31.0)	63.5 (29.8)
No. of samples, (%)	96 (96.0)	54 (53.5)	35 (34.7)	25 (24.8)	72 (71.3)
ECMO blood sampling day, mean (SD)	1.4 (1.2)	8.8 (1.5)	15.9 (1.3)	23 (1.7)	20 (17.3)

D1, ECMO days 1 to 3; D8, ECMO days 8 to 15; D15, ECMO days 15 to 21; D22, ECMO days 21 to 29; ECMO, extracorporeal membrane oxygenation; FXIII, factor XIII; Post, post-ECMO.

When considering the median FXIII activity per patient during ECMO, FXIII deficiency was present in 73.3% of patients (*n* = 74/101) or 73.1% of ECMO runs (*n* = 79/108). Following ECMO removal, FXIII deficiency persisted in 62.5% (*n* = 45/72) and 63.6% (*n* = 49/77) of patients. Among patients with multiple ECMO runs, no difference in FXIII activity was observed between the first and second run (*P* = .607). The prevalence of FXIII deficiency was higher in patients receiving VV ECMO than in those receiving venoarterial ECMO (*n* = 50/74; 67.6%; *P* = .05; Table 1).

C-reactive protein, sequential organ failure assessment score, D-dimer, and disseminated intravascular coagulation score were not associated with the presence of FXIII deficiency. In contrast, FXIII deficiency was significantly more common among patients with viral respiratory tract infections as the underlying condition: 49 of the 74 patients with FXIII deficiency (median FXIII activity during ECMO, 66.2%; *P* = .016) had severe COVID-19–related acute respiratory distress syndrome or influenza. Furthermore, FXIII-deficient patients had a significantly longer ECMO duration compared with those without FXIII deficiency (14 vs 5 days; *P* = .006; Table 1).

**Figure 3:**
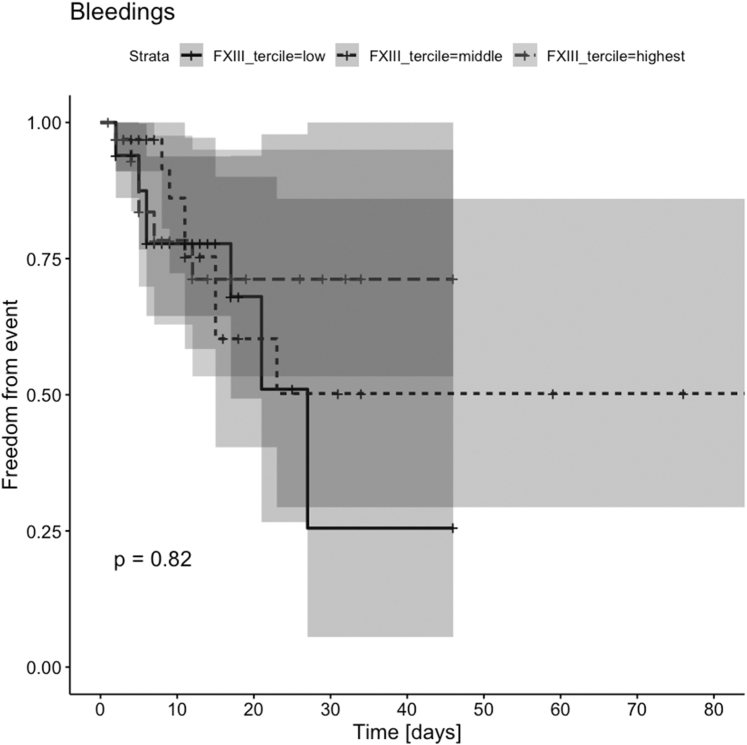
Kaplan–Meier estimates of bleeding-free survival according to factor (F)XIII activity terciles. The Kaplan–Meier curves show bleeding-free survival, divided into 3 groups according to FXIII activity. The shaded areas represent the corresponding CIs. No significant difference in bleeding-free survival was observed between groups (log-rank *P* = .82). Cross marks indicate censored observations.

Thirty-one of 74 patients (41.9%) with FXIII deficiency (median FXIII activity during ECMO) experienced at least 1 bleeding event during ECMO, compared with 3 of 27 patients (11.1%) without FXIII deficiency (*P* = .003). However, there was no significant difference in bleeding-free survival between groups (*P* = .82; Figure 3). Classification of bleeding according to the Bleeding Academic Research Consortium is shown in Table 3. Median FXIII activity during ECMO was significantly associated with bleeding complications in univariate analysis (odds ratio [OR], 0.97; 95% CI, 0.94-0.99; *P* = .003). However, in multivariable logistic analysis adjusted for platelets, age, ECMO duration, and fibrinogen levels, this association was marginally nonsignificant (OR, 0.97; 95% CI, 0.93-1.00; *P* = .07). Minimum FXIII activity (lowest value measured during the entire period of ECMO) was significantly associated with bleeding in both univariate (OR, 0.95; 95% CI, 0.92-0.98; *P* = .001) and multivariable analyses adjusted for the confounders above (OR, 0.97; 95% CI, 0.93-0.99; *P* = .04). In univariate analysis, higher FXIII activity, categorized into tertiles (15%-49%, 50%-69%, and ≥70%), was associated with a significantly lower risk of bleeding, with the odds of bleeding decreasing by 54% per tertile increase (OR, 0.46; 95% CI, 0.25-0.87; *P* = .016). However, this association did not remain statistically significant after adjusting for potential confounders in multivariate analysis (OR, 0.58; 95% CI, 0.31-1.07; *P* = .08). For details, see Table 4.

**Table 3:** Bleeding complications – Bleeding Academic Research Consortium classification.

BARC classification	Overall (*N* = 57)^a^*n* (%)	FXIII deficiency (*n* = 53)^b^*n* (%)	No FXIII deficiency (*n* = 4)^c^*n* (%)
BARC 1	7 (12.3)	5 (9.4)	2 (50.0)
BARC 2	13 (22.8)	13 (24.5)	0 (0.0)
BARC 3a	9 (15.8)	9 (17.0)	0 (0.0)
BARC 3b	23 (40.4)	21 (39.6)	2 (50.0)
BARC 3c	5 (8.8)	5 (9.4)	0 (0.0)
BARC 4	0 (0.0)	0 (0.0)	0 (0.0)
BARC 5	0 (0.0)	0 (0.0)	0 (0.0)

BARC, Bleeding Academic Research Consortium; FXIII, factor XIII.

a All documented bleeding events of all patients.

b All documented bleeding events of all patients with FXIII deficiency.

c All documented bleeding events of all patients without FXIII deficiency.

**Table 4:** Results of multivariate logistic regression of factor XIII activity and bleeding complications (Bleeding Academic Research Consortium 1-3C) adjusted for age, platelets at baseline, and extracorporeal membrane oxygenation type.

Variable	OR	95% CI LB	95% CI UB	*P* value
**Median FXIII activity**	**0.97**	**0.94**	**1.00**	**.04**
Platelets at baseline	1.00	1.00	1.00	.88
Age	1.03	0.98	1.08	.30
ECMO type	4.20	1.22	14.51	.023
**Minimum FXIII activity**	**0.97**	**0.94**	**1.00**	**.023**
Platelets at baseline	1.00	1.00	1.00	.80
Age	1.02	0.98	1.07	.356
ECMO type	2.16	0.69	6.73	.186
**Median FXIII activity tertiles**	**0.58**	**0.31**	**1.07**	**.08**
Platelets at baseline	0.73	1.00	1.00	1.003
Age	1.03	0.98	1.08	.279
ECMO type	2.89	0.96	8.71	.06

The values in bold show the significant correlations.

ECMO, extracorporeal membrane oxygenation; FXIII, factor XIII; LB, lower bound; OR, odds ratio; UB, upper bound.

The risk of thrombosis decreased by 60% with each tertile (OR, 0.40; 95% CI, 0.18-0.88; *P* = .02). In the multivariable model, only FXIII activity (OR, 0.97; 95% CI, 0.93-0.99; *P* = .047) remained significantly associated with thromboembolic events (adjusted for age, platelets, and ECMO duration).

There was no significant difference in ICU or hospital survival between patients with and without FXIII deficiency (ICU survival 66.2% vs 70.4%, *P* = .81; hospital survival 63.5% vs 70.4%, *P* = .639).

This study provides important insights into the prevalence, risk factors, and clinical relevance of FXIII deficiency in adult ECMO patients. In our cohort: (i) two-thirds of patients had FXIII deficiency, (ii) FXIII deficiency was more frequent in VV ECMO, viral respiratory infections, and longer ECMO duration, and (iii) FXIII deficiency was significantly associated with an increased risk of bleeding complications.

We observed a high prevalence of FXIII deficiency, affecting 73% of patients based on the median FXIII activity and 81% based on the lowest FXIII activity. These findings are consistent with previous studies reporting prevalence rates of 69% to 88% based on the lowest FXIII activity during ECMO [8,16]. However, those studies were limited by small sample sizes [16] and potential sampling bias due to their retrospective design [8].

In line with a previous study [8], our data showed that FXIII activity decreased at early time points after ECMO initiation and remained relatively stable over time, with only a slight increase after decannulation. As we did not assess pre-ECMO values, we cannot conclude that the progressive decline was due to ECMO duration alone. In contrast to the aforementioned retrospective study [8], VV ECMO configuration was a significant risk factor for FXIII deficiency in our study, probably driven by longer ECMO duration [10] and a high prevalence of severe COVID-19 acute respiratory distress syndrome, which may promote FXIII deficiency [8,17]. Instead, the initial reduction may be indicative of critical illness and systemic inflammation occurring prior to or at the commencement of ECMO support [18].

In our study, major bleeding occurred in 34% of ECMO patients and was associated with FXIII deficiency. These findings suggest that acquired FXIII deficiency may play an important role in the complex cascade of coagulation changes and dysfunction during ECMO therapy, resulting in increased bleeding morbidity, in agreement with a previous study [8]. However, in multivariate analysis, FXIII activity, adjusted for ECMO duration, age, and platelet count, was borderline nonsignificant for bleeding complications. This probably relates to the fact that there might be a clinically sensible cutoff for the relevance and degree of FXIII activity. Also, our study shows that the risk of bleeding increases by 54% with each tertile of FXIII activity. Although severe bleeding could theoretically reduce FXIII levels, this is unlikely in our cohort due to FXIII’s long half-life and the fact that measurement was taken at the onset of bleeding. Instead, low FXIII levels are more likely to be related to prolonged ECMO, coagulopathy, hemodilution, or impaired synthesis, suggesting a predisposing role in bleeding. While bleeding complications have been identified as important contributors to morbidity and mortality during ECMO, current therapeutic options are limited. FXIII substitution might be an interesting approach in this context. However, our data suggest that higher FXIII levels are associated with thrombotic events, which questions the approach of routinely administering FXIII to bleeding patients. Considering the approach to assess FXIII activity in patients with active bleeding, the turnaround times for FXIII activity determination may limit its usefulness for active interventions. Given the high prevalence of FXIII deficiency, a routine screening approach for all patients appears rational but needs to be balanced against the costs of analysis. A pragmatic approach would be to screen only patients with risk factors, eg, presumed long ECMO duration, VV configuration, and viral infection. To address this knowledge gap, an interventional study assessing FXIII activity and evaluating FXIII substitution in active bleeding is warranted. Of particular interest is whether such an approach could reduce the severity and/or duration of bleeding.

### 3.1 Strengths and limitations

This study provides the first cohort of prospectively collected, consecutive ECMO patients, limiting sampling bias and providing, to some extent, generalizability to other ECMO patients. The main limitations were a single-center design, missing pre-ECMO FXIII levels, measurement of only 1 clotting factor (while multiple deficiencies may have been present), and FXIII assessment at limited time points during ECMO.
